# Anatomical characterization of pTau217 in sporadic AD: Revealing seeding and neurodegeneration within higher‐order cortical circuits

**DOI:** 10.1002/alz70855_104058

**Published:** 2025-12-23

**Authors:** Dibyadeep Datta, Dinara Bolat, Isabella Perone, Denethi Wijegunawardana, Mary Kate Joyce, Christopher H van Dyck, Amy F. T. Arnsten

**Affiliations:** ^1^ Yale University, New Haven, CT, USA; ^2^ Yale School of Medicine, New Haven, CT, USA

## Abstract

**Background:**

Developments in Alzheimer's disease (AD) have revealed a novel fluid‐based pT217‐tau biomarker, in CSF and plasma, that predicts AD prior to cognitive deficits. Understanding the role of pT217‐tau is important in assessing efficacy of novel treatments aimed at early‐stage disease. However, it is unknown why pT217‐tau is effective in predicting brain pathology, as little is known about early‐stage, soluble pT217‐tau brain expression and relationship with markers of neurodegeneration. The etiology of pT217‐tau in aging brains can be probed in the rhesus macaque model of sporadic AD, where perfusion‐fixation allows capture of phosphorylated proteins in their native state. Aging macaques naturally develop tau pathology with the same qualitative pattern and sequence as humans, including initial cortical pathology in layer II entorhinal cortex (ERC) early in aging, and later in layer III dorsolateral prefrontal cortex (dlPFC).

**Method:**

We utilized multi‐label immunofluorescence and immunoelectron‐microscopy to examine the subcellular localization of early‐stage pT217‐tau in ERC and dlPFC of aged macaques with naturally occurring tau pathology.

**Result:**

pT217‐tau is predominantly visualized within dendrites in excitatory neurons and co‐expressed with autophagic vacuolar degeneration (e.g., cleaved HSP70.1), endo‐lysosomal dysfunction, calcium‐activated proteases, markers of neurodegeneration (e.g., cleaved caspase‐3) and dysmorphic mitochondria, indicative of early neurite degeneration. At the subcellular level, pT217‐tau labeling is primarily observed in postsynaptic compartments, accumulating within: 1) dendritic spines on the calcium‐storing smooth endoplasmic reticulum spine apparatus near asymmetric glutamatergic‐like synapses, and 2) in dendritic shafts, where it aggregated on microtubules, often “trapping” endosomes associated with Aβ42. We observed trans‐synaptic pT217‐tau trafficking between neurons within omega‐shaped bodies and endosomes, specifically near excitatory, but not inhibitory synapses.

**Conclusion:**

These data provide the first direct evidence of early‐stage, soluble pT217‐tau aggregating on the microtubules and interfering with endosomal trafficking, appearing to be toxic to dendritic integrity and neuronal function. Furthermore, we find evidence of pT217‐tau trafficking between neurons near synapses to “seed” tau pathology in higher brain circuits, interfacing with the extracellular space to become accessible to CSF and blood. The expression of pT217‐tau in dendrites with early signs of degeneration may help to explain why this tau species can herald future disease.

